# Low-noise time-resolved optical sensing of electromagnetic pulses from petawatt laser-matter interactions

**DOI:** 10.1038/s41598-017-01063-1

**Published:** 2017-04-20

**Authors:** T. S. Robinson, F. Consoli, S. Giltrap, S. J. Eardley, G. S. Hicks, E. J. Ditter, O. Ettlinger, N. H. Stuart, M. Notley, R. De Angelis, Z. Najmudin, R. A. Smith

**Affiliations:** 1 The Blackett Laboratory, Imperial College London, Prince Consort Road, London, SW7 2AZ United Kingdom; 2 ENEA – C.R. Frascati, Dipartimento FSN, Via E. Fermi 45, 00044 Frascati, Italy; 3 Central Laser Facility, STFC Rutherford Appleton Laboratory, Chilton, Didcot, Oxon OX11 0QX United Kingdom

## Abstract

We report on the development and deployment of an optical diagnostic for single-shot measurement of the electric-field components of electromagnetic pulses from high-intensity laser-matter interactions in a high-noise environment. The electro-optic Pockels effect in KDP crystals was used to measure transient electric fields using a geometry easily modifiable for magnetic field detection via Faraday rotation. Using dielectric sensors and an optical fibre-based readout ensures minimal field perturbations compared to conductive probes and greatly limits unwanted electrical pickup between probe and recording system. The device was tested at the Vulcan Petawatt facility with 10^20^ W cm^−2^ peak intensities, the first time such a diagnostic has been used in this regime. The probe crystals were located ~1.25 m from target and did not require direct view of the source plasma. The measured signals compare favourably with previously reported studies from Vulcan, in terms of the maximum measured intra-crystal field of 10.9 kV/m, signal duration and detected frequency content which was found to match the interaction chamber’s horizontal-plane fundamental harmonics of 76 and 101 MHz. Methods for improving the diagnostic for future use are also discussed in detail. Orthogonal optical probes offer a low-noise alternative for direct simultaneous measurement of each vector field component.

## Introduction

Electromagnetic pulses (EMP) are strong, transient electromagnetic fields, typically observed in the radiofrequency (RF) to microwave spectral regions, which can be produced via intense pulsed laser-matter interactions in both the long (nanoseconds to 100 s of picoseconds)^1–11^ and short (picoseconds and shorter)^12–21^ pulse duration regimes, where they can create fields exceeding 1 kV/m. The primary mechanism responsible for generating such high electromagnetic fields in a laser-plasma interaction is typically attributed to ejection of a large flux of energetic ‘hot’ electrons from the plasma followed by a transient ‘slow’ electron return current through the target mount to re-establish quasi-neutrality in the plasma^10, 11, 13, 21^. EMP can be extremely problematic in high-power laser experiments, as EMP signals lasting for hundreds of nanoseconds often adversely affect nearby electronic systems, possibly resulting in loss of data or even permanent damage to high-value equipment. However, RF emission can also be used to infer information about the interaction physics and complement other diagnostic tools when characterising the laser-matter interaction. With the growing number of high-power lasers worldwide^22^ (NIF^23^, OMEGA-EP^24^, Texas Petawatt^25^, Orion^26^, LMJ and PETAL^27^, ELI^28, 29^, Z-Petawatt^30^, Apollon^31, 32^ and more), it is important that we improve our understanding of EMP generation processes from laser-matter interactions for both protecting against its damaging effects (radiation hardening) and to explore its potential uses as a source of fast and intense pulsed electromagnetic fields. The target charging model for EMP generation developed by Poyé *et al*.^20, 21^ has been experimentally validated for short laser pulses with relatively low energies (<80 mJ), however the model’s agreement with experiment is reduced towards the higher end of the investigated energy range, particularly for pulses longer than 0.5 ps, durations which are delivered by many petawatt-scale systems. With the advent ultra-high intensity laser systems, improving theoretical understanding of EMP and our measurement capabilities in the high-energy petawatt-regime has become particularly important.

Conventionally, EMP is measured using a range of conductive antennas or electrical pickup probes connected to recording devices such as oscilloscopes, including but not limited to B-dot and D-dot probes (sensitive to the time derivative of the ***B*** *-* and ***D***-fields) and Moebius loops^33^ (sensitive to magnetic fields). However, these diagnostics all require electrical signals to be transported from the point of measurement to oscilloscopes via conductive cables, and conductive probes can locally perturb the electric fields one is attempting to characterise. Even with shielding, conductive cables can pick up considerable amounts of additional EMP ‘noise’ signal - which might completely obscure the measurement of a particular field component by the probe - making the data difficult to interpret due to very low signal-to-noise ratios. This issue is particularly pronounced on experiments using high-energy picosecond laser pulses. A solution to these problems is to employ a fundamentally different physical process to transform local electric fields into a useful low-noise signal that can be transported to a remote recording instrument with high fidelity. Here we describe the use of the electro-optic effect^34^ for probing EMP; this is suitable for operation in vacuum, uses dielectric crystalline sensors to minimise local disturbance to the field, and is inherently resistant to electrical noise as any ‘long’ distance signal transport can be done via optical fibre. Hence, in terms of electrical shielding, only an effective Faraday cage is required to protect a remote oscilloscope and light-detectors from the general EMP background. This task can be simplified considerably by using sufficiently long fibres to completely remove the detection apparatus from the laser facility’s high-intensity target area, with the caveat that any multi-mode fibres are short enough such that the modal dispersion is less than the time resolution of the optical detectors. As petawatt laser-matter interaction experiments with the current generation of picosecond lasers are inherently single-shot, it is not feasible to reduce noise via statistical methods. Hence, low-noise absolute measurements with minimum disturbance to the original fields are of great interest.

## Methods

A multi-axis optical EMP diagnostic was built as part of an experimental campaign at the Vulcan Petawatt facility (STFC Rutherford Appleton Laboratory) investigating the interaction of intense laser pulses with thin foil and optically levitated^35^ targets. The results from the levitated, mass-limited targets will be discussed in a separate article, currently in preparation. The electric-field components of the EMP were detected on a single-shot basis via the Pockels effect in KDP crystals manufactured for longitudinal Pockels cell modulators. We note that the diagnostic could also be converted to measure magnetic fields by simply exchanging the electro-optic crystals with a magneto-optic medium, such as a high Verdet constant glass.

**Figure 1:**
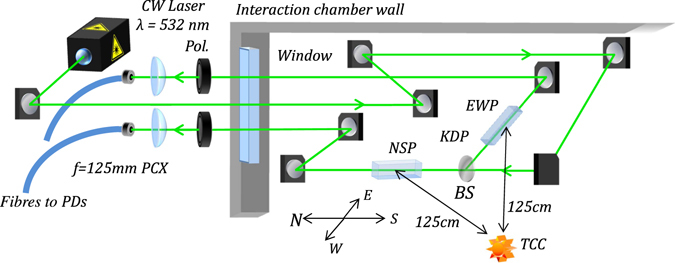
Layout of the optical EMP diagnostic in the Vulcan Petawatt interaction chamber, located near the North-East corner of the 2 × 4.6 × 2.2 m steel vacuum vessel. Compass directions are shown for reference. The laser beam was split between the two directional probes using a 50/50 beam-splitter (BS). In our prototype diagnostic, f = 125 mm plano-convex (PCX) lenses were used to focus into the fibre for simplicity. A vertical direction probe was also built, but did not provide useful data due to some movement of the chamber under vacuum, hence only the East-West and North-South probes (EWP and NSP) were used, with crystals 1.25 m from target chamber centre (TCC).

The Pockels effect scales linearly with applied electric field, hence it is well-suited to absolute field measurements based on use of sensors with well-known dielectric material properties. In our system, the field-induced time-varying polarisation changes on a ~500 mW linearly polarised continuous-wave (CW) 532 nm laser beam, split between orthogonally orientated KDP crystals, were detected as intensity modulations via the inclusion of adjustable transmissive linear polarisers placed in the beam-line after each crystal. This technique is known as *polarisation state modulation*, and has been previously applied to **E**-field measurement in many different contexts^36–48^. It has also been used inversely to measure the electro-optic properties of materials by applying a known field^49^. Here, the use of an intra-cavity frequency-doubled Nd:YAG laser with Type I phase-matching ensured a stable, linearly polarised optical probe source. After transmission through individual KDP crystal “field sensors” and downstream polarisers, the probe beams were focused and guided through 200 µm core diameter step-index multi-mode optical fibres to a Faraday cage housing a 500 MHz oscilloscope (Tektronix DPO4054) and photodetectors on the far side of the target area, approximately 9 m from the source plasma (~8 m of fibre). As the polarisers modulating the light intensity preceded the fibre injection stage, polarisation-maintaining fibres were unnecessary. The diagnostic architecture is shown in Fig. 1. The fibre outputs were directly attached to a set of 1 ns rise-time Thorlabs DET10A photodiodes (PDs) to maximise light collection by the active photosensor region and ensure robust optical alignment. A null channel with an identical photodiode was also attached to monitor any electrical noise pickup within the Faraday cage or fibre fluorescence.

The KDP crystals used in the diagnostic were designed for use with longitudinal electric fields, as in KDP first-order relative retardations are only produced by fields along the optic axis^37^. This was tested by applying transverse electric fields to the crystal via two parallel electrodes fed by a high-voltage pulse driver unit (Kentech PBG1), from which the vectorial selectivity of the crystals was determined to be >35 dB, such that any errors on our measurements from Vulcan due to transverse field effects can be considered negligible. The crystals were rhomboidal with lengths, widths and heights of 25, 13 and 9 mm respectively, with end-faces cut at Brewster’s angle to eliminate “ghost” signals from internal reflections. The use of a longitudinal configuration with the laser beam directed along the optic axis is beneficial as it allows single field components to be measured with discrete crystals, and potential recovery of the full 3D field vector using a set of 3 orthogonal crystals. The phase retardation due to a longitudinal electric field *E*_*l*_ within a crystal of length *L* results in a polarisation rotation Δ*θ* (in radians) given by

1 $${\rm{\Delta }}\theta =\frac{2\pi }{{\lambda }_{0}}{n}_{0}^{3}{r}_{63}L{E}_{l}$$

where *n*_0_ is the crystal’s linear refractive index, *r*_63_ is the electro-optic permittivity and *λ*_0_ is the laser wavelength^37^. Hence, the electric field values are given by

2 $${E}_{l}=\frac{{\rm{\Delta }}\theta {\lambda }_{0}}{2\pi {n}_{0}^{3}{r}_{63}L}$$

The experimental polarisation rotation can be determined by comparison of the “null” voltage values with zero-field in the early time section of each voltage trace before the high-intensity laser irradiated a target. As the polarisers were set such that with zero-field, the voltage readings were half of their maximum value, the fractions of PD voltages *V*_*i*_ relative to the mean zero-field voltage *V*_*0*_ was used to calculate rotation angles for a given voltage value using the relation Δ*θ*_*i*_ = ½ arcsin(*V*_*i*_ */V*_*0*_ − 1). This system is thus sensitive to electric fields of both polarity; fractions of *V*_*i*_/*V*_*0*_ = 1, 0, 2 correspond to respective angles of Δ*θ*_*i*_ = 0°, −45°, +45°. The time resolution of the diagnostic was limited by the 1 ns rise time of the photodiodes, which set an upper bound on the resolvable signal frequencies; the quarter-period of the highest measurable frequency is equal to the rise time, yielding an upper frequency limit of 250 MHz. This measurement bandwidth is considerably lower than the bandwidths of state-of-the-art oscilloscopes and photodetectors (~100 GHz), however the issue may easily be resolved by using faster but more expensive optical sensors and readout systems.

To the best of our knowledge, this is the first time such a diagnostic has been used to measure EMP from petawatt-regime laser-matter interactions. Other work has attempted to optically sense magnetic fields generated by laser-plasma interaction^9^. A similar, independently developed, system to that described in this letter was used by Consoli *et al*. for EMP detection from long-pulse (~3 ns FWHM, focused intensity of ~4 × 10^14^ W cm^−2^) laser-plasma interactions^1^; this diagnostic used a different electro-optic crystal (BSO), laser wavelength and setup, with the sensor crystal placed 85 mm from the target. In the work reported here, the crystals were both placed 1.25 m from the target, with no direct line of sight to mitigate against unwanted detection of x-rays or direct charged particles, positioned with their optic axes along orthogonal axes of the interaction chamber. The ability to detect EMP so far away from the target is useful, as diagnostic access near to the target is typically very limited in high-intensity interaction experiments.

While the work of Consoli *et al*. demonstrated the efficient use of electro-optic EMP measurement techniques in the nanosecond regime^1^, the work described here is the first demonstration of absolute measurement of EMP fields by electro-optic methods in a petawatt-class laser-plasma experiment, using a laser providing hundreds of joules of energy. These are the type of experiments where EMP is observed to be most intense^20^, leading to well-known difficulties in obtaining reliable measurements of EMP fields within the vacuum chamber and also creating greater risk to equipment. The region of parameter space accessed during this experiment is significantly different to that of earlier work with nanosecond lasers; with intensities over a million times higher, the interaction physics is considerably altered, with relativistic effects resulting in additional heating mechanisms (resonant absorption^50, 51^, ***j*** × ***B*** heating^52, 53^ and Brunel heating^54^) and influencing electron transport or blow-off processes. Due to these factors, it is very important to prove that electro-optic techniques can be successfully applied in the petawatt regime, which is most applicable to experiments driven by relativistically intense, highly-energetic, laser systems such as PETAL, Apollon^31^ or the ELI facilities.

**Figure 2:**
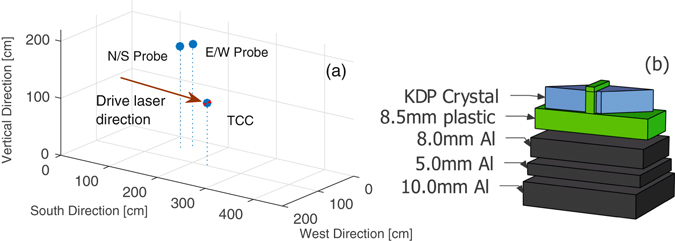
(**a**) Cartesian coordinate plot depicting the location of the KDP crystals within the chamber. The origin is defined here as the bottom North-East corner of the Vulcan Petawatt interaction chamber. Target Chamber Centre (TCC), where targets were located, is also shown for comparison. (**b**) Simplified schematic of the crystal mounts, where the middle two aluminium layers enable fine adjustment and the plastic insulates the crystals from surrounding metals. The aluminium plates were in direct contact with each other, but are shown to be slightly separated here for easier viewing.

Defining the bottom North-East corner of the chamber as the origin of a Cartesian coordinate system (with units in centimetres), the North-South and East-West probe crystals were located at coordinates (56, 142, 181) and (37, 153, 181) respectively, as shown in Fig. 2a.

The crystal mounts consisted of three aluminium plates of 8, 5 and 10 mm thicknesses enabling fine alignment adjustments, shown in Fig. 2b. An additional 8.5 mm-thick plastic layer immediately below each crystal ensured there was no direct contact between crystals and conductive materials. The plate assemblies were attached to a 10 mm-thick stainless steel optical breadboard; these all obscured the direct line of sight between crystals and target, providing significant shielding from direct X-rays and fast particles. The 10 mm breadboard was attached via steel posts to another steel breadboard, with a separation of **~**12 cm. This helps to reduce the X-ray flux incident onto the crystals (which should follow an inverse-square law), as electrons freed by X-ray photons on the surfaces of electro-optic crystal are thought to contribute to the total **E**-field across it^1^. Without shielding, this potential noise contribution could appear to be a low-frequency EMP component, despite not actually coming from the RF emission. However, while providing shielding from X-rays and charged particles, the presence of nearby conductive material results in some local perturbation of the field^1, 7^. KDP is a good choice of crystal for this application, as it possesses a reasonably high electro-optic coefficient of *r*_*63*_ = 9.7 pm/V^55^, and does not scintillate strongly in the absence of impurity ions^56^, so we can neglect X-ray-induced scintillation as a source of unwanted “non-electric field driven” intensity changes. Furthermore, by using a free-space setup within the interaction chamber and placing the fibre-injection optics outside, the coupling of optical self-emission from the target and any scintillation from the crystals into the fibres is minimised, and can be resolved as a prompt event coincident with the arrival of the drive laser, distinct from the longer timescale chamber ring-down.

Isomorphs of dihydrogen phosphates, which include KDP, have been shown to possess clamped electro-optic *r*_*63*_ coefficients that are practically independent of laser wavelength across their transparency ranges^57–59^ and flat frequency responses in the high-frequency (zero strain) limit up to driver field frequencies of 500 MHz^57^, and later ~10 GHz^60^. These are therefore ideal for the diagnostic’s broad frequency range, which could resolve signals between 500 kHz to 250 MHz, determined by the optical detectors’ time-resolution and the chosen temporal measurement window. This is related to the piezo-electric and photo-elastic vibrational behaviour of the crystal; at high frequencies (above ~100 kHz), the crystal motion is inhibited by inertia, yielding the *r*_*63*_ value at zero strain^57^. Hence, in the high-frequency limit of the Pockels effect, KDP has the desirable property of a near-ideal flat frequency response, essential for accurate measurement of EMPs that typically contain a broad frequency spectrum and multiple temporal structures. Even in the low frequency (zero-stress) limit (flat below 20 kHz^57^), *r*_*63*_ = 10.7 pm/V^55^, hence the change in electro-optic coefficient will only result in a small error in determination of the applied **E**-field. The transition region between constant stress and constant strain limits lies between ~20–100 kHz, in which there is larger variation in *r*_*63*_, which must be considered if measuring signals at high precision in this low frequency range.

However, there are also some disadvantages to this diagnostic architecture: it cannot be easily moved within the chamber without realignment, and small movements of the chamber between atmospheric pressures and vacuum could result in some loss of alignment, as the fibre coupling optics were not physically attached to the chamber. As a result, the vertical probe beamline lost alignment upon pumping down the chamber in our proof of concept system, hence only field components in the floor plane were measured, the so-called North-South and East-West chamber modes. This problem could be mitigated via all-fibre delivery into and out of the target chamber, with only the laser and detection system placed externally.

## Results

**Figure 3:**
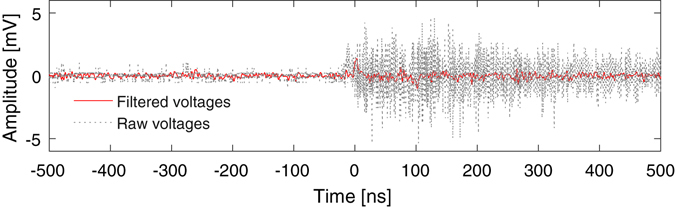
The electrical noise measured by the reference channel both with and without a 70^th^ order Blackman-Harris numerical low-pass filter with 250 MHz cut-off frequency applied. The detected noise has frequency content which mostly exceeds the resolvable frequency range of the diagnostic, and hence can be effectively reduced with a low-pass filter.

The experimental results presented in this article are from a 269 nm thick Parylene-N plastic foil target (1.1 g/cm^3^ mass density), with a laser pulse of duration 1.7 ps, centre wavelength 1054 nm and 386 J delivered to target (227 TW) and focused to a 4.7 × 5.5 µm FWHM spot, with 32.8% of the energy in the FWHM, yielding a peak intensity of 4.8 × 10^20^ W cm^−2^. Due to space constraints we were unable to remove the detectors and oscilloscope entirely from the target area, but we provided shielding of them by using a Faraday cage; however, the few-millivolt EMP-induced ‘noise’ signals on the null photodiode channel were considerably smaller (by ~3 orders of magnitude) than those detected from an end-terminated cable inside the interaction chamber attached to another oscilloscope housed in a Faraday cage close to the experiment (~1 m from the chamber), which could exceed a volt in amplitude. The cable was identical in length to those connected to other (conductive) EMP probes, and was used to provide information on the ‘noise’ contribution of fields coupling directly to the cable and oscilloscope, for comparison to the noise pickup by the electro-optic setup. This information demonstrates the high relative noise-immunity of the electro-optic setup in comparison to conventional methods. Furthermore, much of this unwanted electrical noise was at higher frequencies beyond the diagnostic’s measurement range (as limited by the oscilloscope and photodetectors, which had respective 3 dB points of 500 MHz and 250 MHz), approximately between 0.8–1.2 GHz, and therefore most likely an artefact of the oscilloscope electronics coupling directly with EMP. This allowed the vast majority of the noise to be removed by application of a numerical low-pass filter (finite impulse response 70^th^ order Blackman-Harris window filter with a 250 MHz cut-off frequency), as shown in Fig. 3, such that the noise level before and after the arrival of the main laser pulse is approximately the same.

### Time-resolved field measurements

**Figure 4:**
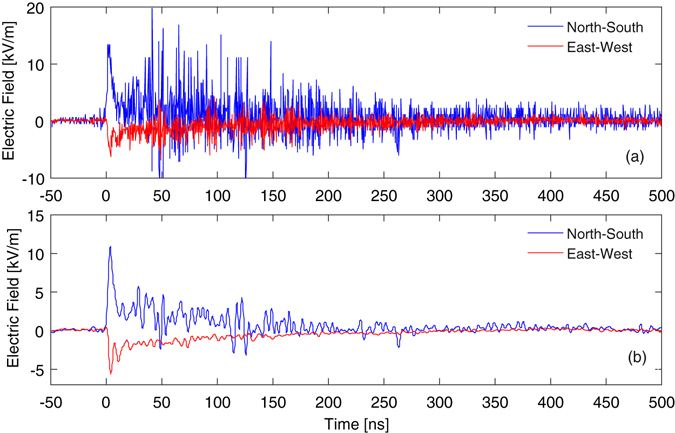
Temporal electric field behaviour (**a**) calculated using the raw voltage data and (**b**) with a low-pass frequency filter applied to remove high-frequency electrical noise, and the contribution to the initial peak by optical self-emission coupled into the optical fibres subtracted. The time axes have been shifted such that *t* = 0 corresponds to the arrival of the 227 TW drive laser pulse on target. A 70^th^ order Blackman-Harris window filter with a cut-off frequency of 250 MHz was chosen to minimize side-lobes and hence time-domain artefacts.

The optical diagnostic successfully measured the EMP’s electric field components within the interaction chamber in both North-South (N/S) and East-West (E/W) directions; the temporal electric field evolutions are shown with and without a numerical low-pass filter applied in Fig. 4a and b respectively. In both probing axes, the EMP signals consisted of rapidly rising peaks of 4.3 ± 0.2 ns (N/S) and 4.0 ± 0.2 ns (E/W) FWHM durations, followed by a decaying oscillation, with reversal of the N/S field direction after ~50 ns. The observed decay in field amplitudes is thought to be due to energy radiated out of the chamber through various diagnostic ports and dielectric windows^7, 8^. The finite impulse response 70^th^ order Blackman-Harris window filter with a 250 MHz cut-off frequency (corresponding to the detector bandwidth) was chosen in order to minimise numerical side-lobes, and hence undesirable time-domain Fourier artefacts arising from frequency domain filtering.

A maximum field in the crystal of 10.9 kV/m was measured with the N/S probe within the resolvable frequency range of the sensor system, with a smaller field component of 5.5 kV/m measured along the E/W axis. Both measurements were made at a distance of 1.25 ± 0.01 m from the plasma. The fields decayed to the observable background noise level after ~500 ns. This is consistent with predicted electric field strengths within the Vulcan Petawatt target chamber calculated by Mead *et al*. to be 7.2 kV/m and 16.5 kV/m in the E/W and N/S directions respectively, based on the idea that the main contribution to EMP is driven by electrons ejected from the plasma striking the interaction chamber wall, causing it to resonate with a dominant N/S mode^13^. The measured values are for fields within the dielectric crystals, hence one expects the vacuum field strengths to be higher and thus closer to Mead’s predicted field strengths. Otherwise, weaker field strengths might be due to a smaller hot electron flux striking the rear chamber wall during the experiment. It should be noted that the maximum field in the time-domain may be larger, as EMP has been reported previously to contain components up to several GHz, which are not detectable by the photodetector or oscilloscope used here, but would be visible to a high time resolution system employing the Pockels effect. However, the difference is likely to be small, as can be seen from the very minor change in maximum electric field between unfiltered and filtered Fig. 4a and b, but it is important to note that this measurement does not provide reliable information regarding the content of the high frequency signal components.

From background test shots with the probe laser inactive, the small peaks on each channel (including the ‘null’ noise reference) arising from coupling of self-emission from the plasma into the optical fibres are consistently the same size on a given shot, however there is some small variation between shots due to differences in drive laser pulse energies. Hence, by processing the data from the electrical noise reference channel on each shot exactly as if it were a ‘real’ electric field measurement, the apparent contribution of the self-emission to the initial peaks can be determined and accounted for. This method gives apparent **E**-fields of +1.9 kV/m and +0.5 kV/m on the N/S and E/W signals respectively, which have been subtracted from the initial peaks in Fig. 4b. Due to the single-shot nature of high-energy petawatt laser-plasma experiments, it is generally not possible to gather extensive statistics for a given measurement to reduce uncertainty. The driver-field frequency response of KDP is flat in the range of resolvable frequencies, hence the uncertainty on the measured field is predominantly due to the instrument noise on each measurement including oscilloscope white and sampling noise and residual EMP coupling to the electronics. This can be determined by again examining the apparent **E**-fields on the noise reference channel to obtain the instrument noise within the time-window of interest, thus incorporating all of the aforementioned noise contributions; with maximum noise amplitudes of ±0.9 (N/S) and ±0.3 kV/m (E/W), this corresponds to ±8.2% and ±5.4% uncertainties on the N/S and E/W mode peaks respectively. For weaker signals, the percentage error correspondingly increases, with a value of ±50% on the minimum measurable field values based on a minimum acceptable signal-to-noise ratio of 2. Temperature variation is another potential source of uncertainty in electro-optic measurement techniques, with phase retardation varying by −0.18%/K for KDP at room temperature^37^. In our case however, the measurement was robust as the temperature within the Vulcan chamber is stable within <1 °C.

### EMP spectral analysis

**Figure 5:**
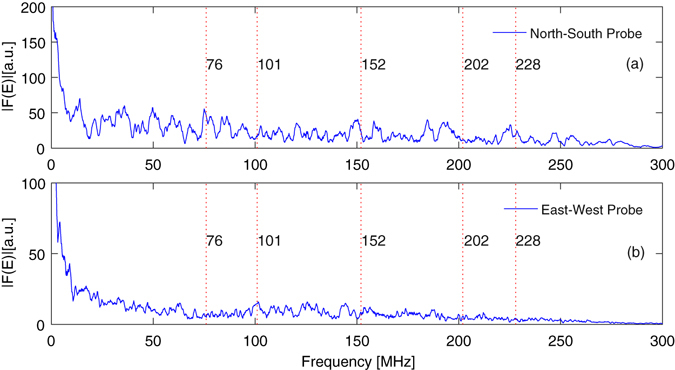
Frequency spectra of the EMP with the 250 MHz cut-off low-pass filter applied from (**a**) the North-South and (**b**) East-West probes. Several peaks are visible in the spectra, with some corresponding extremely well with the theoretical fundamental chamber resonances and their harmonics (marked on the plot). The large quantity of conductive materials within the interaction chamber is expected to allow several other resonances to be supported, to which we attribute the presence of the other spectral peaks. A 1^st^ degree Savitzky-Golay filter was applied in the frequency domain for noise-reduction.

The Vulcan interaction chamber is a conductive cuboid, with 2, 2.2 and 4.6 m separations between steel walls in the East-West, vertical, and North-South dimensions respectively; treating the chamber as a simple parallel cavity resonator supporting Transverse Electric (TE) modes, the fundamental resonance frequencies are set by the chamber geometry. These correspond to resonances of 76, 82 and 101 MHz in the E/W, vertical and N/S dimensions respectively^13^. It is important to note that these frequencies are expected based on an *empty* interaction chamber, and thus are likely subject to some considerable modification by in-chamber hardware including metallic optical tables and optomechanics. The optical diagnostic detected the fundamental resonances of the interaction chamber at 76 ± 4 and 101 ± 2 MHz, although not on the expected probe channels. Frequency content at 149 ± 5 and 225 ± 6 MHz was also measured, matching the predicted 2^nd^ and 3^rd^ harmonics of the E/W mode (152 and 228 MHz), albeit in the N/S probing direction; these are summarised in Table 1. A spectrum is shown in Fig. 5, obtained by Fast-Fourier Transform of the low-pass filtered time-domain E-field data.

**Table 1:** Frequencies of the expected harmonics and detected spectral peaks.

Expected Frequencies/MHz	Measured Frequencies/MHz		
	*EO Ch1* (*North-South*)	*EO Ch2* (*East-West*)	*D-Dot*
76^E-W1^	76 ± 4	Not detected	Not detected
101^N-S1^	102.7 ± 0.6	101 ± 2	Not detected
152^E-W2^	149 ± 5	Not detected	148.5 ± 4.5
202^N-S2^	Not detected	Not detected	Not a sharp peak
228^E-W3^	225 ± 6	Not detected	Not a sharp peak

^E-W^ or ^N-S^ signify the mode axis, ^m=1,2,3^ gives the harmonic order.

Interestingly, the expected E/W fundamental mode frequencies were detected more strongly by the N/S probe, which also seems to have a narrow peak at 102.7 ± 0.6 MHz, near the N/S fundamental of 101 MHz. The peak field magnitude detected by this probe was approximately twice that of the orthogonal field component, and some of the frequency content in this dimension is likely to have been up- or down-shifted to other frequencies by the presence of numerous metal objects throughout the chamber (such as the silver main focussing parabola optics mounts, breadboards and a range of other diagnostics), which allow multiple additional resonances and their harmonics to be supported^7^, potentially with similar spectral amplitudes to the fundamental cavity modes as observed in our case. Furthermore, the rapid transient discharge current through the target holder, as described by the Poyé model^20, 21^, will result in electromagnetic field emission at a specific range of frequencies not necessarily corresponding to the chamber fundamentals, which in turn may also be frequency shifted.

**Figure 6:**
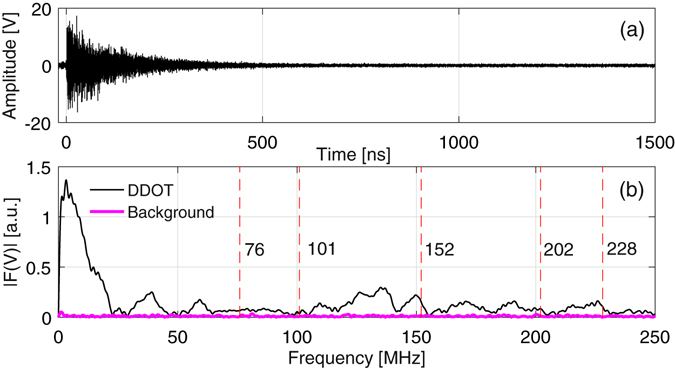
(**a**) Time domain signal measured by the D-Dot probe. (**b**) Spectrum in the 0–250 MHz frequency range for associated EMP and background noise signals. Harmonics corresponding to those theoretically expected in chamber and listed in Table 1 are outlined here by dashed red vertical lines.

In Fig. 6a we show the signal acquired with a 12.5 GHz Tektronix DPO71254C oscilloscope, from a customised commercial D-Dot conductive probe, placed within the experimental chamber at the coordinates West: 72 cm, South: 4 cm, Vertical: 160 cm, of the reference system in Fig. 2, behind a ~10 cm thick silver-coated glass mirror with respect to the target. This protects it from direct line of sight to the majority of X-rays and particles emitted from the target. The probe is a customised version of the Prodyn AD-80(R), with 5.5 GHz of 3 dB bandwidth. It was equipped with a balun (model BIB-170G), having 150 kHz–10 GHz of 3 dB bandwidth and leading to common mode rejection >20 dB. The probe has a dual-dipole structure with high rejection to common mode disturbances. It was set in a vertical plane (edge on to the target) such that both dipoles were exposed to residual hard X-rays coming from the laser-plasma that would, to first order, generate a common mode effect on the probe, thus cancelling or at least reducing the common mode. The spectrum was obtained by applying the Fast Fourier Transform to the first 600 ns of the signal of Fig. 6a and it is shown in Fig. 6b in the 0–250 MHz range, to compare these results with those already discussed for the electro-optical measurements. In Fig. 6b an estimation of the background noise spectrum is also shown. This was achieved by applying the FFT to the signal in Fig. 6a on the (600 ns, 1200 ns) interval, where the EMP contribution had already ceased. The spectrum of the EMP signal detected by the D-DOT is well beyond the background level for most of the observed frequency range. Harmonics corresponding to those theoretically expected within the chamber and listed in Table 1 are outlined with dashed red lines. Information on measurements associated with these resonances is reported on the same Table. Good correspondence with both the expected theoretical values and electro-optic measurements is found for the 148.5 MHz contribution; some agreement can be observed also for both the 202 MHz and the 228 MHz harmonics. It is generally difficult to make reliable comparisons between different EMP diagnostics unless they are in identical locations; the presence of numerous metal objects within the target chamber results in complex EMP field topologies^7^, such that field strengths and relative strengths of the spectral components can vary greatly between different positions within the chamber. Moreover, diagnostics of different nature often have different detection efficiencies, in terms of bandwidth and polarisation. These issues are further compounded by inherent shot-to-shot energy variation and movement of diagnostics and other metallic objects between shots, such that recreating the same conditions if placing probes in identical positions on different shots is non-trivial in practice. A detailed discussion of this topic, including comparisons between the electro-optic probes, the D-DOT probe and other EMP diagnostics deployed on this experiment will be addressed in a separate publication.

**Figure 7:**
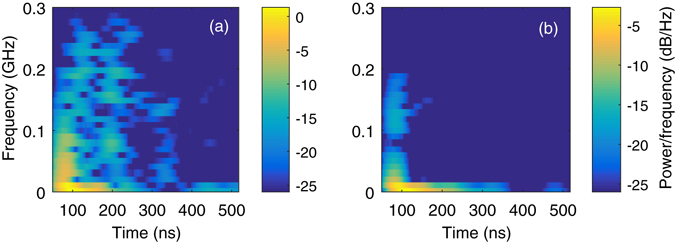
Spectrograms depicting the temporal changes in the spectral behaviour of the low-pass filtered electric field traces from shot #29 for (**a**) the N/S probe and (**b**) the E/W probe. As the channels measure orthogonal fields, different frequencies are strongly detected in the early time region, some of which match the chamber fundamental modes.

Time-frequency analysis was also carried out on the electro-optic data using the short-time Fourier transform technique spectrogram MATLAB function; this provides insight into the temporal behaviour of the various frequency components and energy transfer between different chamber modes. Spectrograms for each measurement channel are shown in Fig. 7. The different chamber fundamental modes are apparent in the early time regions of the spectrograms from the bright regions separated in frequency space, however these quickly decay below the minimum measurable field level of the E/W probe (Fig. 7b), consistent with the much weaker signals measured along that axis.

## Discussion

The minimum intra-crystal electric field observable with this new diagnostic is set by the minimum polarisation rotation-induced intensity change resolvable above the experimental noise; this was determined to be 1.8 kV/m for the N/S channel and 0.6 kV/m for the E/W, based on a minimum acceptable signal-to-noise ratio of 2, as analysis of the noise reference channel yielded maximum noise features of 0.9 and 0.3 kV/m for the N/S and E/W probing channels. This could be effectively improved by increasing the laser power reaching the photodiodes, either by optimising coupling efficiency into the optical fibres, or by simply using a more powerful probe laser. Hence, a given polarisation rotation will result in a larger measured voltage change relative to the noise, allowing smaller fields to be measured, thus increasing the diagnostic’s dynamic range. As the polarisers in the diagnostic were set such that half of the CW laser power was transmitted with no electric field applied to the crystals, the maximum measurable field *E*_*max*_ is set by the field required to rotate the laser polarisation by ±45° and can be calculated using Eq. (2), yielding |*E*_*max*_| = 79.2 kV/m using *r*_*63*_ = 9.7 × 10^−12^ m/V^55^, *n*_*0*_ = 532 nm, *L* = 25 mm and *n*_*0*_ = 1.514 at 532 nm. Hence, the useful dynamic range is currently ~45, limited by electrical noise pickup by the detection setup. This could be improved by a factor of ~5 before necessitating use of a more advanced recording system with a greater than bit-depth than is typical for fast 8-bit oscilloscopes operating in single shot mode.

It is important to note that the above values represent the electric fields *inside the crystals* rather than the vacuum fields within the chamber. It is possible to calibrate the crystals to external fields using a TEM cell setup^61, 62^, however doing this for relatively large longitudinal probe crystals is not trivial, requiring custom apparatus. Typically, we are most interested in *relative* EMP measurements between shots while varying a parameter such as target thickness or pulse energy, however an approximation for the radiated vacuum fields can be calculated using the method described by Massey *et al*.^37^; this analysis approximates rectangular crystals as ellipsoids of revolution about the optic axis and only requires knowledge of the crystal aspect ratio *a* (the ratio of the longest and shortest axes) and dielectric constant along the optic axis *ε*. The analysis uses the Poisson equation to estimate **E-**fields under the electro-quasistatic approximation, where times of interest *t*_*1*_ (here the period of a frequency component) must be much longer than the time *t*_*2*_ required for an electromagnetic wave to propagate at the velocity *c/n* (*c* is the speed of light, *n* is the refractive index of the propagation medium) over the largest length of the system^63^, in our case the crystal length *L*. Since the largest frequency that the electro-optic diagnostic can measure well is 250 MHz, the smallest *t*_*1*_ is 4 ns, therefore the condition is satisfied as *t*_*2*_ = 382 ps for a propagating 250 MHz **E**-field. The vacuum field strength *E*_*0*_ is related to the intra-crystal field *E*_*crys*_ by

3 $${E}_{0}\approx \frac{{E}_{crys}({D}_{3}^{-1}+\varepsilon -1)}{{D}_{3}^{-1}}$$

where *D*_*3*_ is the depolarisation factor^64^ for the optic axis, the major axis of the ellipsoid of revolution given by

4 $${D}_{3}={b}^{-1}\{\frac{1}{2}a{b}^{-1/2}\,\mathrm{ln}[\frac{a+{b}^{1/2}}{a-{b}^{1/2}}]-1\}$$

where we have defined *b* = (*a* ^*2*^ − 1) for convenience. For the crystals used here, *a* = 2.78 and *ε* = 21^65^, yielding *D*_*3*_ = 0.11924 and hence and an approximate conversion relation between vacuum and intra-crystal fields of *E*_*0*_ ≈ 3.38*E*_*crys*_, such that we can thus estimate the maximum radiated field strengths to be approximately 37 and 19 kV/m in the N/S and E/W axes. This can be compared to an estimated free-space to crystal coupling parameter of ~2.84 based on measurements using a pulsed open-capacitor setup, where the crystals placed between the two parallel capacitor plates were subject to a ~9 kV pulse amplitude longitudinal electric field. This coupling parameter gives corresponding vacuum fields of approximately 31 and 16 kV/m, in reasonably good agreement with the values predicted from electro-quasistatic theory. The high voltage pulses were produced using a Kentech PBG1 Pockels cell driver, with Indium Tin Oxide (ITO) coated PET sheets, separated by 10 cm, acting as transparent planar electrodes. The electrode dimensions were 250 × 200 × 0.2 mm, such that the parallel plates were large compared to the crystal dimensions to avoid any fringe-field effects and maximise the uniformity of the longitudinal calibration field.

It should be noted that if the fields are sufficiently strong to either extinguish the laser or double the voltage measured by the photodiodes, there is a potential ambiguity in the measurements as fields greater than |*E*_*max*_| will induce polarisation rotations greater than ±45°, which will result in a change in laser intensity with the opposite sign to that which is expected. Hence, it is essential that users are aware of this ambiguity if the change in photodiode voltage approaches the magnitude of the zero-field voltage (*V*_*i*_ → *2V*_*0*_ or 0) while running the diagnostic. This problem can be compensated for by changing the electro-optic crystal to obtain different *r*_*63*_ and *n*_*0*_ values, or alternatively by changing the laser wavelength; by selecting a lower *r*_*63*_ crystal or a longer wavelength, the polarisation rotation for a given electric field strength can be reduced, allowing the maximum measurable field value to be increased before the aforementioned ambiguity becomes significant. The crystal length is also a key parameter for selecting the range of measurable fields, with increasing length leading to higher linear sensitivities.

There are some diagnostic design improvements that could be implemented relatively easily on our proof of principle device. We provide a summary here for researchers wishing to recreate an optical EMP diagnostic. Firstly, delivery of the laser light into and out of the chamber via optical fibre would allow the device to be more mobile within the interaction chamber, thus minimising any space constraints with simultaneous use of other diagnostics. The dynamic range of the diagnostic can be significantly improved via two methods: reducing electrical noise, and increasing the incident laser power on the diodes through both optimised fibre injection and higher initial laser power by a factor of ~5 before necessitating the use of a >8 bit detection system. For optimum noise reduction, we strongly recommend complete removal of detectors and oscilloscopes from the target area into a Faraday cage in a well-shielded room; as there can be no electrical noise pickup from the optical fibres, this should allow significant signal-to-noise ratio improvements compared to conventional “all electrical” EMP diagnostics. Note that modal dispersion should be considered if using multimode fibres over ‘long’ distances, such that the maximum possible time-delay between modes is smaller than photodiode rise times.

The diagnostic’s time-resolution and bandwidth could be improved by simply using faster photodiodes and oscilloscopes, though this is primarily a matter of cost rather than technical capability. However, it should be noted that the propagation time through the crystals can also limit the ultimate time resolution, as the beam effectively sees the ‘average’ field over this time frame, hence it is unnecessary to use photodiodes with shorter rise times than the crystal transit time. For the 2.5 cm long KDP crystals, the transit time for 532 nm light is ~126 ps (3.5 GHz bandwidth), hence the time resolution could be increased by almost an order of magnitude by only replacing the photodiodes and oscilloscope. The fastest commercially available photodiodes for visible light have rise times of ~9 ps and 45 GHz bandwidths (Newport Model 1014), therefore a crystal length of 1.8 mm would be necessary to match ~9 ps time resolution, at the expense of reduced sensitivity. If the crystal type was changed for operation in the mid infrared (1550 nm), just outside of KDP’s transparency range, 100 GHz photodetectors could be purchased (Finisar XPDV412xR). Deuterated KD*P crystals are transparent between 200–2100 nm and, as dihydrogen phosphate isomorphs, offer similarly flat frequency-response properties as KDP but with higher electro-optic coefficients. It should be noted that the fastest oscilloscopes are limited to bandwidths of ~100 GHz, hence a hypothetical diagnostic using such a setup would be approaching the limit of available electronic measurement technology. The quarter-period of a 100 GHz photodiode is 2.5 ps, so assuming the rise-time matches the quarter period of the maximum resolvable frequency component, a KD*P crystal thickness of 520 µm (commonly manufactured) would be necessary to maintain time resolution. However, the sensitivity of such a short crystal to electric fields would be considerably reduced (by 98% compared to a 25 mm crystal), and hence would only be suited to measurement of very strong electric fields. Furthermore, increasing the bandwidth of oscilloscopes also typically increases the intrinsic noise, resulting in poor signal-to-noise ratios. An electro-optic probing system for EMP using two complementary diagnostics can be considered; one very fast but with low sensitivity, and another with far higher sensitivity but correspondingly reduced bandwidth. Alternatively, a non-oscilloscope based readout system could be considered for very high time resolution applications, for example through use of a streak camera, at the expense of a reduced temporal measurement window.

Finally, a useful design change would be to replace all metal components in the diagnostic architecture with dielectric counterparts. This would minimise local electric field perturbations from the optomechanical setup for optimal measurement of the original fields at the diagnostic’s location. Implementation of all the aforementioned upgrades would become costly when attempting to operate at frequencies approaching 100 GHz and hence only realistic for large national facilities if such high frequency measurement was essential. However, the system described here demonstrates the intrinsic robustness and promise of the electro-optic EMP measurement principle.

## Conclusions

We have described the first electro-optic measurement of EMP from petawatt-regime short-pulse laser-plasma interactions with low electrical noise background and no requirement for direct-view or close proximity to the target. In light of new ultra-high intensity laser systems, such as PETAL, Apollon and the various ELI lasers, coming into operation in the near future, it is important that we can demonstrate the applicability of devices with high inherent noise-immunity capable of measuring EMP in this specific regime of extreme laser-plasma physics. Not only is the EMP particularly problematic in experiments using such high-intensities (such as ion acceleration or fast-ignition inertial fusion schemes), this area is where interest is at a maximum from within this research community. Maximum field components of 10.9 ± 0.9 and 5.5 ± 0.3 kV/m were measured within the crystals at a distance of 1.25 m from the plasma (with external fields estimated to be ~31–37 and ~16–19 kV/m, depending on whether the experimental or theoretical conversion parameter is used), and the fundamental chamber resonances at 76 and 101 MHz and some of their harmonics were detected, in good agreement with previous EMP studies at Vulcan using conductive probes and theoretical models. The optical diagnostic is inherently highly resistant to the electrical noise that plagues conventional EMP measurement techniques using conductive probes and cables, and hence provides an attractive alternative for monitoring EMP at high-power laser facilities. Relatively easily attainable performance increases in resolution, bandwidth and noise reduction are possible by increasing the CW probe laser power, using faster photodetectors, changing the electro-optic crystal lengths and improved electrical shielding or remote operation of the detection apparatus. The diagnostic could also be made more mobile by introducing fibre transport into and out of the interaction chamber, a desirable property as diagnostic access around target-chamber centre is often very limited. Furthermore, the diagnostic setup could be adapted for magnetic field detection by replacing the electro-optic KDP crystals with a suitable magneto-optic medium such as a high-Verdet constant glass.
